# Confirmation of herbicide resistance mutations Trp574Leu, ΔG210, and EPSPS gene amplification and control of multiple herbicide-resistant Palmer amaranth (*Amaranthus palmeri*) with chlorimuron-ethyl, fomesafen, and glyphosate

**DOI:** 10.1371/journal.pone.0214458

**Published:** 2019-03-26

**Authors:** Douglas J. Spaunhorst, Haozhen Nie, James R. Todd, Julie M. Young, Bryan G. Young, William G. Johnson

**Affiliations:** 1 United States Department of Agriculture, Agricultural Research Service, Sugarcane Research Unit, Houma, Louisiana, United States of America; 2 Department of Botany and Plant Pathology, Purdue University, West Lafayette, Indiana, United States of America; University of Guelph, CANADA

## Abstract

Herbicide-resistant weeds, especially Palmer amaranth (*Amaranthus palmeri* S. Watson), are problematic in row-crop producing areas of the United States. The objectives of this study were to determine if chlorimuron-ethyl, fomesafen, and glyphosate applied separately and in mixtures control *A*. *palmeri* and confirm the presence of various genotypes surviving two- and three-way herbicide mixtures. Fifteen percent of *A*. *palmeri* treated with the three-way herbicide mixture survived. Mixing fomesafen with chlorimuron-ethyl or fomesafen with glyphosate to create a two-way mixture reduced *A*. *palmeri* survival 22 to 24% and 60 to 62% more than glyphosate and chlorimuron-ethyl alone, respectively. Previously characterized mutations associated with *A*. *palmeri* survival to chlorimuron-ethyl, fomesafen, and glyphosate *Trp*_*574*_*Leu*, a missing glycine codon at position 210 of the *PPX2L* gene (*ΔG210*), and 5-enolpyruvylshikimate-3-phosphase synthase (*EPSPS*) gene amplification; respectively, were present in surviving plants. However, 37% of plants treated with chlorimuron-ethyl did not contain heterozygous or homozygous alleles for the *Trp*_*574*_*Leu* mutation, suggesting alternative genotypes contributed to plant survival. All surviving *A*. *palmeri* treated with fomesafen or glyphosate possessed genotypes previously documented to confer resistance. Indiana soybean [*Glycine max* (L.) Merr] fields infested with *A*. *palmeri* possessed diverse genotypes and herbicide surviving plants are likely to produce seed and spread if alternative control measures are not implemented.

## Introduction

Herbicides are the backbone for weed control in large-scale agricultural production systems in developed countries. However, poor herbicide stewardship has resulted in the loss of several of these herbicides [1,2]. Glyphosate is a non-selective herbicide that was developed in 1970 and used extensively in orchards to control weeds. In 1996, transgenic soybean [*Glycine max* (L.) Merr.] cultivars resistant to glyphosate were released for commercial use [3]. Since 2003, glyphosate-resistant *G*. *max* varieties have been planted annually to more than 80% of *G*. *max* hectares in the US [4]. This level of glyphosate use has contributed to numerous weed control failures due to resistance evolution [5]. Palmer amaranth (*Amaranthus palmeri* S. Watson) is a notable problematic weed that has evolved resistance to glyphosate. In Georgia, glyphosate applied at five times the typical field use rate resulted in 46% *A*. *palmeri* control at 4 WAT [6]. The Macon County, GA population was the first confirmed case in 2004 where *A*. *palmeri* evolved resistance to glyphosate in the US. Plants from the same Georgia population possessed 100 or more 5-enolpyruvylshikimate-3-phosphase synthase (*EPSPS*) copies, a mechanism that confers resistance to glyphosate in *A*. *palmeri* [7].

Herbicides applied post-emergence (POST) to crops with minimal to no crop injury that result in a high level of weed control are greatly desired. Fomesafen is a protoporphyrinogen oxidase (PPO)- inhibiting herbicide that is applied POST for control of *A*. *palmeri* in *G*. *max*. *G*. *max* is transiently injured by fomesafen, but after 14 days displays marginal phytotoxic effects when applied within label recommendations [8]. *G*. *max* plants metabolize the diphenylether family of herbicides, which includes acifluorfen and fomesafen, by cleavage of the diphenylether bond into non-toxic conjugates [9]. *A*. *palmeri* resistant to fomesafen was reported in Arkansas in 2011 [10]. Plants that survived contained a glycine amino acid deletion (*ΔG210*) in *PPX2L* [10]. Currently, the *ΔG210* deletion is the only known mechanism to confer PPO-inhibitor resistance in common waterhemp [*Amaranthus tuberculatus* (Moq.) J. D. Sauer], a species similar to *A*. *palmeri* [11,12]. More recently, two additional mutations in *A*. *palmeri*, *R98M* and *R98G*, which conferred resistance to PPO-inhibiting herbicides [13].

Chlorimuron-ethyl is in the sulfonylurea family of acetolactase synthase (ALS) inhibiting herbicides. Herbicides inhibiting ALS are applied at very low use rates and bind to an active site only found in plants and microorganisms. However, various mutations in the ALS active site compromise herbicide binding affinity [14]. Currently, four amino acid substitutions at four positions on the ALS gene confer *A*. *palmeri* resistance to ALS-inhibiting herbicides [15,16]. The *Trp*_*574*_*Leu* amino acid substitution is a common mutation reported to cause resistance to the ALS inhibitors in *A*. *palmeri*, *A*. *tuberculatus*, redroot pigweed (*Amaranthus retroflexus* L.), Powell amaranth (*Amaranthus powellii*), and kochia [*Bassia scoparia* (L.) A. J. Scott] [17–21]. *A*. *palmeri* resistant to ALS-inhibiting herbicides was first reported in Kansas in 1993 and has since been documented in 12 other states [22].

Use of prepackaged mixtures that contain more than one herbicide site of action has become popular since the spread of glyphosate-resistant (GR) weeds [23]. Herbicide mixtures control problematic weeds such as *A*. *tuberculatus*, giant ragweed [*Ambrosia trifida* (L.)], and *A*. *palmeri*; however, some herbicide mixtures negatively affect herbicide efficacy [24–26]. One study reported up to 16% reduction in *A*. *palmeri* control with 420 g ha^-1^ of fomesafen mixed with 420 g ha^-1^ of glyphosate at 4 WAT [27]. However, 9 g ha^-1^ of chlorimuron mixed with 420 g ha^-1^ of glyphosate increased *A*. *palmeri* control 10% more than 420 g ha^-1^ of glyphosate applied alone at 4 WAT. A variety of weed species response to mixtures of 240 g ha^-1^ of fomesafen plus glyphosate applied at rates from 280 to 1,120 g ha^-1^ showed that mixtures did not reduce broadleaf signalgrass [*Urochloa platyphylla* (Munro ex C. Wright) R.D. Webster], johnsongrass [*Sorghum halepense* (L.) Pers.], hemp sesbania [*Sesbania herbacea* (Mill.) McVaugh] or pitted morningglory [*Ipomoea lacunosa* (L.)] fresh weight at 4 WAT [28]. However, herbicide antagonism resulted when the two systemic herbicides, 8.7 g ha^-1^ of chlorimuron plus 1,120 g ha^-1^ of glyphosate, were mixed and further increased *I*. *lacunosa* fresh weight by 24% at 4 WAT. In a different study, 17 g ha^-1^ of cloransulam-methyl plus 280 g ha^-1^ of fomesafen resulted in 23 and 71% more prickly sida [*Sida spinosa* (L.)] control than 280 g ha^-1^ of fomesafen and 17 g ha^-1^ of cloransulam-methyl applied separately, respectively [29].

Herbicide mixtures are generally more effective in providing consistent weed control and also control a larger spectrum of weed species than a single herbicide [26,28]. However, many commonly used herbicide mixtures in *G*. *max* contain active ingredients that *A*. *palmeri* has evolved resistance to. Herbicide mixtures have resulted in moderate control of multiple herbicide-resistant (HR) *A*. *palmeri*. A mixture of glyphosate plus thifensulfuron plus atrazine resulted in 55% control of a putative glyphosate, ALS, and atrazine-resistant *A*. *palmeri* population [15]. A putative *A*. *palmeri* population resistant to atrazine and mesotrione was identified in a seed corn production field in 2010 in Nebraska [30]. In the previous study the authors reported 7 and 58% control with 560 g ha^-1^ of atrazine and 106 g ha^-1^ of mesotrione, respectively; however, mixing the herbicides resulted in 41 to 92% more control than atrazine or mesotrione applied separately [30].

Failure of commonly applied herbicides in *G*. *max* production systems to control *A*. *palmeri* in Indiana with resistance to glyphosate and potentially chlorimuron-ethyl and fomesafen led to this research. Previous research reported some *A*. *palmeri* biotypes collected in Indiana were GR and two biotypes exhibited increased tolerance to 2,4-D amine [31]. In the same study, the authors reported complete *A*. *palmeri* control when plants were treated with a mixture of glyphosate plus 2,4-D choline [31]. The first objective of this experiment was to evaluate *A*. *palmeri* response to two- and three-way herbicide mixtures. The second objective was to identify the genotypes of surviving herbicide-treated plants and confirm the presence of various genotypes surviving two- and three-way herbicide mixtures.

## Materials and methods

### Seed collection

In late-summer of 2013 seeds from suspected HR *A*. *palmeri* were harvested from female plants from agricultural production fields infested with *A*. *palmeri*, dried in the greenhouse for two weeks, and threshed [32]. Threshed seeds were stored in a cooler at 4 C for 2 yr before seeded in the greenhouse. The locations where *A*. *palmeri* seeds were collected are presented in Table 1. Permissions were granted to collect weed seeds and the study did not involve endangered or protected species.

**Table 1:** Location of Indiana fields where Palmer amaranth (*Amaranthus palmeri* S. Watson) seeds were collected in 2013 and the herbicide resistance profile assigned for each county based on *A*. *palmeri* injury (0–100%) to chlorimuron-ethyl (39 g ai ha^-1^), fomesafen (1,026 g ai ha^-1^), and glyphosate (2,500 g ae ha^-1^) in the initial screen for herbicide resistance experiment.

County^a^	Coordinates		Herbicide resistance profile^b^								
			Chlorimuron-ethyl			Fomesafen			Glyphosate		
	Latitude	Longitude	R	MR	S	R	MR	S	R	MR	S
Washington	38.75°N	86.06°W			X			X			X
Daviess	38.85°N	87.08°W		X			X			X	
Cass	40.86°N	86.20°W	X					X	X		
Unknown^c^^d^	NA	NA	──	──	──	──	──	──	──	──	──

^a^ *A*. *palmeri* seeds from suspected herbicide-resistant plants were collected from agricultural production fields infested with *A*. *palmeri*. *A*. *palmeri* seeds from the unknown county were purchased from Azlin Seed Service, Leland, MS. The site location of the unknown population was not available.

^b^ A herbicide resistance profile was assigned to counties based on visible injury of surviving *A*. *palmeri* in the initial screen for herbicide resistance experiment: R, resistant (0–40% injury); MR, moderately resistant (41–80% injury); and S, susceptible (81–100% injury).

^c^ *A*. *palmeri* from the unknown county was not evaluated in the initial screen for herbicide resistance experiment, but was included as a glyphosate-sensitive check.

^d^ A total of twenty plants from the unknown county were tested for amplified 5-enolpyruvylshikimate-3-phosphase synthase and *ΔG210* mutations that confer resistance to glyphosate and fomesafen, respectively, and no plants possessed either herbicide resistant trait. The *Trp*_*574*_*Leu* amino acid substitution mutation that confers resistance to acetolactase synthase inhibiting herbicides was identified in *A*. *palmeri* from the unknown location.

### Initial screen for herbicide resistance

To determine the sensitivity of *A*. *palmeri* to chlorimuron-ethyl, fomesafen, and glyphosate herbicides an initial screen for herbicide resistance was conducted. Based on results from the preliminary screen, *A*. *palmeri* from Washington County were susceptible (81–100% injury) to chlorimuron-ethyl, fomesafen, and glyphsoate applied individually; however, *A*. *palmeri* from Daviess County were moderately resistant (41–80% injury) to the aforementioned herbicides (Table 1). *A*. *palmeri* individuals from Cass County were susceptible to fomesafen, but were resistant (0–40% injury) to chlorimuron-ethyl and glyphosate applied separately (Table 1). Approximately 300 *A*. *palmeri* seeds from each county were germinated on 28 cm by 55 cm by 2 cm, 200 square plastic-plug trays using potting medium and covered with clear plastic lids for 40 h in the greenhouse. A single plant at the two true-leaf stage was transplanted into a 10-cm by 10-cm pot filled with equal proportions of soil, sand, and potting medium (Redi-Mix, Sun-Gro Redi-Earth Plug and Seedling Mix, Sun-Gro Horticulture, Bellevue, WA) and fertilized every two weeks (Miracle-Gro Water Soluble All Purpose Plant Food [24–8–16], Scotts Miracle-Gro Products Inc., Marysville, OH). Greenhouse temperatures were maintained from 23 to 30 C and plants were exposed to supplemental lighting with a 16 hour photoperiod. Herbicides representing each site of action were selected based on herbicide use patterns in Indiana. When plants were approximately 8-cm tall (6- to 8-leaf stage), 39 g ai ha^-1^ of chlorimuron-ethyl (trade name: Classic DuPont Crop Protection, Wilmington, DE), 1,026 g ai ha^-1^ of fomesafen (trade name: Flexstar, Syngenta Crop Protection, Inc., Greensboro, NC), and 2,500 g ae ha^-1^ of glyphosate (trade name: Touchdown Hi-Tech, Syngenta Crop Protection, Inc., Greensboro, NC) were applied separately. Ten plants were treated to every herbicide treatment from each collection location (Washington, Daviess, and Cass Counties) and a non-treated check was included for comparison (10 replications*3 collection locations*4 treatments *n* = 120). All treatments included 0.25% (v/v) non-ionic surfactant (trade name: Activator 90, Loveland Products, Greeley, CO) plus 2.9 kg ai ha^-1^ of ammonium sulfate (trade name: N-Pak AMS 3.4L, Winfield Solutions, St. Paul, MN). Spray applications were made inside an enclosed track-spray chamber with a single 8002E nozzle (TeeJet, Spraying Systems Co., Wheaton, IL) and a carrier volume of 140 L ha^-1^ at a pressure of 207 kPa. Plants were returned to the greenhouse after treatment application.

### Whole-plant greenhouse assay

A whole-plant greenhouse assay was conducted to determine susceptibility of *A*. *palmeri* to chlorimuron-ethyl, fomesafen, and glyphosate applied separately and in all possible mixtures. *A*. *palmeri* seed germination, transplanting, and herbicide rates were same as previously mentioned in the initial screen for herbicide resistance section. A list of herbicide treatments, herbicide resistance mechanism(s) tested, all possible genotype combinations, and number of genotype combinations identified for each herbicide treatment are presented in Table 2. The rates previously mentioned represented three times the commonly applied field use rates and were chosen based off discriminating doses from preliminary greenhouse studies. The experiment was conducted as a randomized complete block design and the experiment was repeated. To ensure rare HR mutations were identified from the Daviess County population, suspected to harbor plants with multiple HR traits based on results from the initial screen for herbicide resistance study, 20 plants were exposed to each herbicide treatment. The replicate size was 10 for all other treatments and collection locations (Washington, Cass, and unknown). Prior to herbicide treatment plants were sorted by height. The tallest plants (average height: 9-cm; 8- to 10-leaf stage) were arranged in replication one and shorter plants (average height: 6.5-cm; 6- to 8-leaf stage) were placed in replication 10 for Washington, Cass, and Unknown collection locations and replication 20 for Daviess County. Sprayer settings were identical to those mentioned previously in the initial screen for resistance study and plants were returned to the greenhouse after treatment application.

**Table 2:** List of herbicide treatments, herbicide resistance mechanism(s), and genotypes of Palmer amaranth (*Amaranthus palmeri* S. Watson) treated to chlorimuron-ethyl, fomesafen, and glyphosate separately and in all possible combinations in the greenhouse^a^.

Herbicide treatment	Rate	Resistance mechanism(s) tested^b^	Total possible genotypes	Genotypes identified in surviving herbicide treated plants^c^
	g ai or ae ha^-1^		────────── # ──────────	
Chlorimuron-ethyl	39	*Trp*_*574*_*Leu*	3	3
Fomesafen	1,026	*ΔG210*	3	2
Glyphosate	2,500	Amplified *EPSPS*	2	1
Chlorimuron-ethyl plus fomesafen	39 plus 1,026	*Trp*_*574*_*Leu* and *ΔG210*	9	2
Chlorimuron-ethyl plus glyphosate	39 plus 2,500	*Trp*_*574*_*Leu* and Amplified *EPSPS*	6	3
Fomesafen plus glyphosate	1,026 plus 2,500	*ΔG210* and amplified *EPSPS*	6	3
Chlorimuron-ethyl plus fomesafen plus glyphosate	39 plus 1,026 plus 2,500	*Trp*_*574*_*Leu*, *ΔG210*, and amplified *EPSPS*	18	7

^a^ Abbreviations: EPSPS, 5-enolpyruvylshikimate-3-phosphase synthase;

^b^ Alleles for *Trp*_*574*_*Leu* and *ΔG210* resistance mechanisms were heterozygous, homozygous-resistant, or wild type for a total of three possible genotypes. Two genotypes were possible for *EPSPS* copy number. Plants with ten or more *EPSPS* copies possessed the *EPSPS* amplified genotype and plants with *EPSPS* copy number from 1 to 9 were denoted as the wild type.

^c^ Confirmed herbicide-resistant genotypes using molecular screening assays.

### Molecular screen for herbicide-resistant mutations *Trp*_*574*_*Leu*, *ΔG210*, and *EPSPS* gene amplification

Newly emerged leaf tissue from the same plants in the whole-plant greenhouse assay were removed before herbicide treatment from each plant and placed in an individual 2 mL centrifuge tube. Centrifuge tubes were labeled by county, herbicide treatment, and replication; therefore, a genotype was assigned to each plant based on real-time quantitative polymerase chain reaction (qPCR) results. Leaf material was stored at -80 C until DNA was extracted. Genomic DNA was extracted with the use of a modified cetyl trimethylammonium bromide (CTAB) method [33]. DNA extractions totaled 350 per experimental run. Plants treated with a single herbicide in the whole-plant greenhouse assay experiment were tested for the single HR mutation of interest. Plants treated with multiple herbicides were tested for multiple herbicide resistance mutations. *EPSPS* gene amplification was determined as previously described by Gaines et al. [7]. To detect the presence or absence of the *ΔG210* mutation, the same allele-specific probes were used as described by Giacomini et al. [13]. Allele-specific probes determined whether a plant was homozygous-resistant, heterozygous, or wild type for the *ΔG210* mutation. A TaqMan probe was developed to test for the presence of the *Trp*_*574*_*Leu* mutation that is often present in ALS-resistant *Amaranthus* species [16–18,21]. The following probe 5’-ATCGATCTTCCAATTGAA-3’ (AHOJE43_VIC) was used to identify homozygous-resistant or heterozygous plants harboring the *Trp*_*574*_*Leu* mutation, while the probe 5’-TCGATCTTCCCATTGAA-3’ (AHOJE43_FAM) detected wild type plants. The forward and reverse primers used to flank TGG to TTG were 5’-CCGGTTAAAATCATGCTCTTGAACAAT-3’ and 5’-TGTGCCCGGTTAGCTTTGTAAA-3’, respectively. Manager software (Bio-Rad Laboratories) was used for data analysis, which reported the relative florescence units of each allele. Equation one was used to express data generated from the qPCR as normalized relative fluorescence units (nRFU) [34] where:

$$\frac{RFUA1}{\left[RFUA1+RFUA2+u(NTC)\right]}=nRFUA1$$ (1)

The ratio of nRFU of *PPX2L* to nRFU of Δ*PPX2L* generated from the qPCR determined whether plants were homozygous-resistant, heterozygous, or wild type. Similarly, the ratio of nRFU of *Trp*_*574*_ to nRFU of *Leu*_*574*_ determined if plants were homozygous-resistant, heterozygous, or wild type. The *Trp*_*574*_*Leu* mutation, at the time the study was conducted, was a common ALS mutation responsible for plant survival to ALS-inhibiting herbicides.

### Data collection

At 21 days after treatment (DAT), each plant was rated as alive (green tissue or red-colored stems were present) or dead (green tissue or red-colored stems were absent). The genotype of surviving plants were determined using qPCR as previously discussed, and the frequency of each genotype for tested resistance mechanism(s) were tabulated and are presented in Table 3.

**Table 3:** Genotypes(s) and frequency of surviving Palmer amaranth (*Amaranthus palmeri* S. Watson) plants treated to chlorimuron-ethyl, fomesafen, and glyphosate separately and in all possible combinations^a^.

Herbicide treatment^b^	Resistance mechanism(s) tested	Genotype(s)^c^	Survival frequency
			#
Chlorimuron-ethyl	*Trp*_*574*_*Leu*	Heterozygous	30
		Homozygous	4
		Wild type	37
Fomesafen	*ΔG210*	Heterozygous	6
		Homozygous	3
Glyphosate	Amplified *EPSPS* copy number	≥10 EPSPS copies	33
Chlorimuron-ethyl plus fomesafen	*Trp*_*574*_*Leu* and *ΔG210*	Heterozygous and heterozygous	7
		Homozygous and homozygous	1
		Wild type and heterozygous	1
Chlorimuron-ethyl plus glyphosate	*Trp*_*574*_*Leu* and amplified *EPSPS* copy number	Heterozygous and ≥10 EPSPS copies	7
		Homozygous and ≥10 EPSPS copies	7
		Wild type and ≥10 EPSPS copies	14
Fomesafen plus glyphosate	*ΔG210* and amplified *EPSPS* copy number	Heterozygous and ≥10 EPSPS copies	7
		Homozygous and ≥10 EPSPS copies	4
Chlorimuron-ethyl plus fomesafen plus glyphosate	*Trp*_*574*_*Leu*, *ΔG210*, and amplified *EPSPS* copy number	Heterozygous, heterozygous, and ≥10 EPSPS copies	3
		Heterozygous, homozygous, and ≥10 EPSPS copies	4
		Heterozygous, wild type, and ≥10 EPSPS copies	1
		Heterozygous, homozygous, and wild type	1
		Homozygous, homozygous, and ≥10 EPSPS copies	1
		Wild type, heterozygous, and ≥10 EPSPS copies	4
		Wild type, wild type, and ≥10 EPSPS copies	1

^a^ Abbreviations: *EPSPS*, 5-enolpyruvylshikimate-3-phosphase synthase.

^b^ A total of 100 *A*. *palmeri* plants were exposed to each herbicide treatment. The frequency of surviving herbicide treated plants and their respective genotype for each resistance mechanism are presented.

^c^ Alleles for *Trp*_*574*_*Leu* and *ΔG210* resistance mechanisms were heterozygous, homozygous-resistant, or wild type for a total of three possible genotypes. Two genotypes were possible for *EPSPS* copy number. Plants with ten or more *EPSPS* copies possessed the *EPSPS* amplified genotype and plants with *EPSPS* copy number from 1 to 9 were denoted as the wild type.

### Statistical analysis

Typical statistical assumptions of normal distribution and equal variance were not met; therefore, the Box-Cox transformation was applied to identify an appropriate transformation to normalize survival data. The Box-Cox transformation produced a lambda value of -0.25 and statistical assumptions were revalidate using the inverse square root and logarithmic transformations. The inverse square root and logarithmic transformations did not improve normal distribution or equal variance assumptions; therefore, survival was compared by herbicide treatment using PROC GLIMMIX in SAS (v. 9.3 SAS Institute, 100 SAS Campus Drive, Cary, NC) and nontransformed means are reported. Fixed effects included herbicide treatment and run; replication and collection location were random effects. Means were separated using an adjusted Tukey test at the 0.05 level of significance. Data were pooled across experimental run due to no significant run effect.

## Results

The study showed *A*. *palmeri* individuals survived two- and three-way herbicide mixtures commonly applied to control *A*. *palmeri* in *G*. *max*. *A*. *palmeri* survival was influenced by herbicide treatment and was greatest when plants were treated with chlorimuorn-ethyl alone (Table 4). Other researchers have shown poor GR *A*. *palmeri* control with chlorimuron-ethyl [35]. Glyphosate and fomesafen applied separately failed to eradicate all plants; however, glyphosate and fomesafen applied separately reduced *A*. *palmeri* survival 38 and 62% more than chlorimuron-ethyl, respectively (Table 4). One method to alleviate glyphosate-induced weed shifts is to mix glyphosate with an herbicide that targets an alternative site of action [36]. However, combining chlorimuron-ethyl with glyphosate did not reduce *A*. *palmeri* survival when compared to glyphosate alone (Table 4). Other researchers reported that glyphosate and chlorimuron-ethyl mixtures enhanced *A*. *palmeri* absorption of ^14^C-chlorimuron-ethyl by 16% when compared to chlorimuron-ethyl alone; however the herbicide mixture increased *A*. *palmeri* control no more than 8% when compared to the chlorimuron-ethyl and glyphosate applied separately [27]. Fomesafen plus glyphosate or fomesafen plus chlorimuron-ethyl mixtures did not increase or decrease *A*. *palmeri* survival when compared to fomesafen applied alone (Table 4). Nandula et al. [37] reported 60 g ha^-1^ of flumiclorac, a PPO-inhibiting herbicide, mixed with 840 g ha^-1^ of glyphosate antagonized GR *A*. *palmeri* control 23% or more and reduced glyphosate translocation by 19 and 36% at 1 and 2 DAT, respectively. In contrast, other research has shown fomesafen plus glyphosate mixtures are beneficial for control of other weed species. Research showed 30 and 68% less fresh weight biomass in *S*. *herbacea* and *I*. *lacunosa*, respectively, at 28 DAT with mixtures of 240 g ha^-1^ of fomesafen plus 1,120 g ha^-1^ of glyphosate when compared to 1,120 g ha^-1^ of glyphosate alone [28].

**Table 4:** Palmer amaranth (*Amaranthus palmeri* S. Watson) survival to chlorimuron-ethyl, fomesafen, and glyphosate applied separately and in all possible combinations in the greenhouse^a^.

Herbicide treatment^b^	Survival^c^
	%
Chlorimuron-ethyl	71 a
Fomesafen	9 d
Glyphosate	33 b
Chlorimuron-ethyl plus fomesafen	9 d
Chlorimuron-ethyl plus glyphosate	28 bc
Fomesafen plus glyphosate	11 d
Chlorimuron-ethyl plus fomesafen plus glyphosate	15 cd

^a^ A total of 100 *A*. *palmeri* plants were exposed to each herbicide treatment.

^b^ Plants were sprayed at 6.5- to 9-cm in height (6- to 8-true leaves) and evaluated at 21 days after treatment. Plants that survived contained green tissue or red-colored stems.

^c^ Means followed by the same letter are not statistically different (Tukey HSD [0.05]).

To confirm the presence of various genotypes surviving two- and three-way mixtures, surviving plants were genotyped for the HR mutations *Trp*_*574*_*Leu*, *ΔG210*, and amplified *EPSPS* copy number and sprayed with chlorimuron-ethyl, fomesafen, or glyphosate. Genotype data confirmed the herbicide resistance mechanisms *Trp*_*574*_*Leu*, *ΔG210*, and amplified *EPSPS* copy number, previously identified in *A*. *palmeri* in other US states, exist in *A*. *palmeri* collected from Indiana *G*. *max* fields (Table 3). All possible genotypes (heterozygous, homozygous-resistant, and wild-type) for the *Trp*_*574*_*Leu* mutation were present in surviving *A*. *palmeri* plants (Table 3). This result suggests other mechanisms contribute to *A*. *palmeri* survival to chlorimuron-ethyl and that the *Trp*_*574*_*Leu* mutation partially accounted for chlorimuron-ethyl resistance. The *Trp*_*574*_*Leu* mutation is not the only mutation that confers ALS-resistance in *A*. *palmeri*, but is a common point mutation found in *Amaranthus* species that have evolved resistance to ALS-inhibiting herbicides [20,38,39]. In one study, *A*. *palmeri* with a *Ser*_*653*_*Asn* mutation were also resistant to ALS-inhibiting herbicides [16]. Non-target site resistance mechanisms may also be responsible for *A*. *palmeri* survival to chlorimuron-ethyl. A non-target site resistance mechanism resulted in ALS resistance in an *A*. *tuberculatus* population from Illinois [38]. Some land grant universities provide services to screen for mutations associated with herbicide resistance in *A*. *palmeri* and *A*. *tuberculatus*. Therefore, research is needed to investigate additional ALS mutations associated with *A*. *palmeri* survival to chlorimuron-ethyl. Screening *A*. *palmeri* for additional ALS mutations may result in more accurate predictions of *A*. *palmeri* survival to ALS-inhibiting herbicides.

Two genotypes were identified in surviving fomesafen treated *A*. *palmeri* plants (Table 2). Plants harbored heterozygous or homozygous-resistant alleles for *ΔG210*, in fact, there were three more heterozygous than homozygous-resistant plants (Table 3). These data suggested surviving fomesafen-treated *A*. *palmeri* are likely to produce progeny that possess heterozygous and homozygous-resistant *ΔG210* genotypes if alternative control measures are not implemented. In fact, a shift towards more homozygous-resistant than heterozygous genotypes will occur if plants are exposed to repeated treatments of fomesafen and survive to produce seed. Copy number analysis revealed that *EPSPS* gene amplification was present in all plants that survived the glyphosate treatment (Table 3). *A*. *palmeri* survival to glyphosate due to *EPSPS* gene amplification has been rigorously documented in the literature [7,35,40].

## Discussion

*A*. *palmeri* survival to chlorimuron-ethyl varied from plant death to marginal or no injury for plants harboring the *Trp*_*574*_*Leu* genotype, which indicated that the *Trp*_*574*_*Leu* mutation is not an adequate indicator of susceptibility to chlorimuron-ethyl and that alternative genotypes were responsible for *A*. *palmeri* survival. The evolution of GR and PPO-inhibitor resistance in *A*. *palmeri* should be alarming, considering that glyphosate and fomesafen are common herbicides used for POST weed control in GR *G*. *max* cropping systems. Horseweed [*Conyza candensis* (L.) Cronq.], *A*. *tuberculatus*, and *A*. *trifida* are problematic weeds Indiana growers contend with that have evolved resistance to glyphosate. A more alarming discovery was individuals were identified to harbor genes that allow *A*. *palmeri* survival to mixtures of chlorimuron-ethyl, fomesafen, and glyphosate. Although many *A*. *palmeri* in the non-treated check began to initiate inflorescence when the study was terminated at 21 DAT; plants that survived the three-way mixture had not begun to emerge inflorescence by experiment termination. Given the biology of the species, the potential for multiple HR plants to produce HR pollen and seed is likely in surviving plants allowed to continue growth in the field, creating a high risk for rapid multiple resistance evolution within individuals and populations. The obvious contribution of HR gene flow via seeds and pollen to the prevalence of multiple resistance highlights the importance of controlling weed escapes in the field, but also field borders, roadsides, ditch banks [41].

The first case of *A*. *palmeri* resistance evolution to fomesafen was reported in Arkansas [10]. Since then, *A*. *palmeri* resistant to PPO-inhibiting herbicides has been reported in Indiana, Illinois, and Tennessee [22]. Many PPO-inhibiting herbicides exhibit pre-emergence (PRE) and POST herbicidal activity. An *A*. *palmeri* biotype from Tennessee was not controlled with a POST fomesafen treatment and was also poorly controlled with soil-applied fomesafen and sulfentrazone treatments [42]. Metabolic resistance to PPO-inhibitor herbicides is another resistance mechanism in addition to resistance conferred by point mutations in *PPX2L*. Treating PPO-resistant *A*. *palmeri* with 1,500 g ha^-1^ of malathion followed by 263 g ha^-1^ of fomesafen 2 hours later reduced plant survival 22% more than fomesafen alone, indicating that fomesafen metabolism was responsible for *A*. *palmeri* survival [43]. Reverting a weed population back to the wild type is unlikely, given that a fitness penalty is not linked to a particular HR trait [44,45]. However, in the case of metabolic resistance evolution to fomesafen, mixing a cytochrome P450 inhibitor (malathion) or GST inhibitor (NBD-Cl; 4-chloro-7-nitrobenzofurazan) with fomesafen can reduce PPO-resistant *A*. *palmeri* survival [43]. Future research is needed to evaluate the efficacy of PPO-inhibiting soil-applied herbicides flumioxazin, saflufenacil, sulfentrazone, and fomesafen on *A*. *palmeri*.

Continued spread of *A*. *palmeri* seed within Indiana is likely to occur, given that the weed is already present in the northern and southern regions. One study reported that *A*. *palmeri* adapted to Arkansas, Mississippi, Missouri, and Nebraska are able to develop and produce copious amounts of seed if introduced to Indiana [46]. The data in this report showed the diversity of *A*. *palmeri* genotypes to documented HR mechanisms and confirmed *A*. *palmeri* survival to three-way herbicide mixtures. Management strategies that include cover crops, cultivation, hand-hoeing, planting *G*. *max* in narrow-rows, and selecting crop cultivars that rapidly canopy are necessary strategies that complement diversified PRE and POST herbicide programs.

## Supporting information

S1 Dataset *A*. *palmeri* injury to chlorimuron-ethyl (0.39 g ai ha^-1^), fomesafen (1,026 g ai ha^-1^), and glyphosate (2,500 g ae ha^-1^) in the initial screen for herbicide resistance experiment. (PDF)

S2 Dataset *A*. *palmeri* survival after treatment to chlorimuron-ethyl (0.39 g ai ha^-1^), fomesafen (1,026 g ai ha^-1^), and glyphosate (2,500 g ae ha^-1^) applied separately and in all possible combinations in the greenhouse. (PDF)
