# Supplementary material for: Confirmation of herbicide resistance mutations Trp574Leu, ΔG210, and EPSPS gene amplification and control of multiple herbicide-resistant Palmer amaranth (Amaranthus palmeri) with chlorimuron-ethyl, fomesafen, and glyphosate
Source: PLoS One. 2019 Mar 26;14(3):e0214458. doi: 10.1371/journal.pone.0214458 (PMC6435131; doi:10.1371/journal.pone.0214458)
Supplement: S1 Dataset — (PDF) [file pone.0214458.s001.pdf]

S1 Dataset. A. palmeri injury to chlorimuron-ethyl (0.39 g ai ha^{-1}), fomesafen (1,026 g ai ha^{-1}), and glyphosate (2,500 g ae ha^{-1}) in the initial screen for herbicide resistance experiment.

County	Rep	Treatment	Injury
Cass	1	chlorimuron-ethyl	0
Cass	2	chlorimuron-ethyl	0
Cass	3	chlorimuron-ethyl	0
Cass	4	chlorimuron-ethyl	0
Cass	5	chlorimuron-ethyl	0
Cass	6	chlorimuron-ethyl	0
Cass	7	chlorimuron-ethyl	0
Cass	8	chlorimuron-ethyl	0
Cass	9	chlorimuron-ethyl	0
Cass	10	chlorimuron-ethyl	0
Cass	1	fomesafen	100
Cass	2	fomesafen	100
Cass	3	fomesafen	100
Cass	4	fomesafen	100
Cass	5	fomesafen	100
Cass	6	fomesafen	100
Cass	7	fomesafen	100
Cass	8	fomesafen	100
Cass	9	fomesafen	100
Cass	10	fomesafen	100
Cass	1	glyphosate	0
Cass	2	glyphosate	10
Cass	3	glyphosate	0
Cass	4	glyphosate	0
Cass	5	glyphosate	0
Cass	6	glyphosate	0
Cass	7	glyphosate	0
Cass	8	glyphosate	10
Cass	9	glyphosate	0
Cass	10	glyphosate	5
Daviess	1	chlorimuron-ethyl	70
Daviess	2	chlorimuron-ethyl	35
Daviess	3	chlorimuron-ethyl	35
Daviess	4	chlorimuron-ethyl	60
Daviess	5	chlorimuron-ethyl	95
Daviess	6	chlorimuron-ethyl	60
Daviess	7	chlorimuron-ethyl	70
Daviess	8	chlorimuron-ethyl	40
Daviess	9	chlorimuron-ethyl	35
Daviess	10	chlorimuron-ethyl	65
Daviess	1	fomesafen	100
Daviess	2	fomesafen	100
Daviess	3	fomesafen	100

Daviess	4	fomesafen	80
Daviess	5	fomesafen	40
Daviess	6	fomesafen	80
Daviess	7	fomesafen	60
Daviess	8	fomesafen	60
Daviess	9	fomesafen	85
Daviess	10	fomesafen	100
Daviess	1	glyphosate	55
Daviess	2	glyphosate	50
Daviess	3	glyphosate	45
Daviess	4	glyphosate	45
Daviess	5	glyphosate	40
Daviess	6	glyphosate	55
Daviess	7	glyphosate	40
Daviess	8	glyphosate	35
Daviess	9	glyphosate	55
Daviess	10	glyphosate	35
Washington	1	chlorimuron-ethyl	65
Washington	2	chlorimuron-ethyl	70
Washington	3	chlorimuron-ethyl	95
Washington	4	chlorimuron-ethyl	65
Washington	5	chlorimuron-ethyl	80
Washington	6	chlorimuron-ethyl	70
Washington	7	chlorimuron-ethyl	80
Washington	8	chlorimuron-ethyl	100
Washington	9	chlorimuron-ethyl	85
Washington	10	chlorimuron-ethyl	90
Washington	1	fomesafen	100
Washington	2	fomesafen	100
Washington	3	fomesafen	100
Washington	4	fomesafen	100
Washington	5	fomesafen	100
Washington	6	fomesafen	100
Washington	7	fomesafen	100
Washington	8	fomesafen	100
Washington	9	fomesafen	100
Washington	10	fomesafen	100
Washington	1	glyphosate	100
Washington	2	glyphosate	100
Washington	3	glyphosate	100
Washington	4	glyphosate	100
Washington	5	glyphosate	100
Washington	6	glyphosate	100
Washington	7	glyphosate	100
Washington	8	glyphosate	100
Washington	9	glyphosate	100
Washington	10	glyphosate	100
