# Supplementary material for: Confirmation of herbicide resistance mutations Trp574Leu, ΔG210, and EPSPS gene amplification and control of multiple herbicide-resistant Palmer amaranth (Amaranthus palmeri) with chlorimuron-ethyl, fomesafen, and glyphosate
Source: PLoS One. 2019 Mar 26;14(3):e0214458. doi: 10.1371/journal.pone.0214458 (PMC6435131; doi:10.1371/journal.pone.0214458)
Supplement: S2 Dataset — (PDF) [file pone.0214458.s002.pdf]

S2 Dataset. A. palmeri survival after treatment to chlorimuron-ethyl (0.39 g ai ha^{-1}), fomesafen (1,026 g ai ha^{-1}), and glyphosate (2,500 g ae ha^{-1}) applied separately and in all possible combinations in the greenhouse.

County	Rep	Run	Treatment	Genotype	Survival
Cass	1	1	Chlorimuron-ethyl	Heterozygote	100
Cass	2	1	Chlorimuron-ethyl	Heterozygote	100
Cass	3	1	Chlorimuron-ethyl	SUS	100
Cass	4	1	Chlorimuron-ethyl	SUS	100
Cass	5	1	Chlorimuron-ethyl	Heterozygote	100
Cass	6	1	Chlorimuron-ethyl	SUS	0
Cass	7	1	Chlorimuron-ethyl	SUS	100
Cass	8	1	Chlorimuron-ethyl	Heterozygote	100
Cass	9	1	Chlorimuron-ethyl	SUS	100
Cass	10	1	Chlorimuron-ethyl	RES	100
Cass	1	2	Chlorimuron-ethyl	Heterozygote	100
Cass	2	2	Chlorimuron-ethyl	Heterozygote	100
Cass	3	2	Chlorimuron-ethyl	Heterozygote	100
Cass	4	2	Chlorimuron-ethyl	Heterozygote	100
Cass	5	2	Chlorimuron-ethyl	RES	100
Cass	6	2	Chlorimuron-ethyl	SUS	100
Cass	7	2	Chlorimuron-ethyl	Heterozygote	100
Cass	8	2	Chlorimuron-ethyl	SUS	100
Cass	9	2	Chlorimuron-ethyl	RES	100
Cass	10	2	Chlorimuron-ethyl	SUS	100
Cass	1	1	Fomesafen	SUS	0
Cass	2	1	Fomesafen	SUS	0
Cass	3	1	Fomesafen	SUS	0
Cass	4	1	Fomesafen	SUS	0
Cass	5	1	Fomesafen	SUS	0
Cass	6	1	Fomesafen	SUS	0
Cass	7	1	Fomesafen	SUS	0
Cass	8	1	Fomesafen	SUS	0
Cass	9	1	Fomesafen	SUS	0
Cass	10	1	Fomesafen	SUS	0
Cass	1	2	Fomesafen	SUS	0
Cass	2	2	Fomesafen	SUS	0
Cass	3	2	Fomesafen	SUS	0
Cass	4	2	Fomesafen	SUS	0
Cass	5	2	Fomesafen	SUS	0
Cass	6	2	Fomesafen	SUS	0
Cass	7	2	Fomesafen	SUS	0
Cass	8	2	Fomesafen	SUS	0
Cass	9	2	Fomesafen	SUS	0
Cass	10	2	Fomesafen	SUS	0
Cass	1	1	Glyphosate	H	100
Cass	2	1	Glyphosate	H	0

Cass	3	1	Glyphosate	H	100
Cass	4	1	Glyphosate	H	100
Cass	5	1	Glyphosate	H	100
Cass	6	1	Glyphosate	H	100
Cass	7	1	Glyphosate	H	100
Cass	8	1	Glyphosate	H	100
Cass	9	1	Glyphosate	.	100
Cass	10	1	Glyphosate	.	100
Cass	1	2	Glyphosate	H	0
Cass	2	2	Glyphosate	H	100
Cass	3	2	Glyphosate	H	100
Cass	4	2	Glyphosate	H	100
Cass	5	2	Glyphosate	H	0
Cass	6	2	Glyphosate	H	100
Cass	7	2	Glyphosate	H	100
Cass	8	2	Glyphosate	H	100
Cass	9	2	Glyphosate	H	100
Cass	10	2	Glyphosate	H	100
Cass	1	1	Chlorimuron-ethyl + fomesafen	HetSUS	0
Cass	2	1	Chlorimuron-ethyl + fomesafen	HetSUS	0
Cass	3	1	Chlorimuron-ethyl + fomesafen	SUSSUS	0
Cass	4	1	Chlorimuron-ethyl + fomesafen	RESSUS	0
Cass	5	1	Chlorimuron-ethyl + fomesafen	HetSUS	0
Cass	6	1	Chlorimuron-ethyl + fomesafen	HetSUS	0
Cass	7	1	Chlorimuron-ethyl + fomesafen	HetSUS	0
Cass	8	1	Chlorimuron-ethyl + fomesafen	SUSSUS	0
Cass	9	1	Chlorimuron-ethyl + fomesafen	SUSSUS	0
Cass	10	1	Chlorimuron-ethyl + fomesafen	HetSUS	0
Cass	1	2	Chlorimuron-ethyl + fomesafen	SUSSUS	0
Cass	2	2	Chlorimuron-ethyl + fomesafen	RESSUS	0
Cass	3	2	Chlorimuron-ethyl + fomesafen	SUSSUS	0
Cass	4	2	Chlorimuron-ethyl + fomesafen	SUSSUS	0
Cass	5	2	Chlorimuron-ethyl + fomesafen	HetSUS	0
Cass	6	2	Chlorimuron-ethyl + fomesafen	HetSUS	0
Cass	7	2	Chlorimuron-ethyl + fomesafen	HetSUS	0
Cass	8	2	Chlorimuron-ethyl + fomesafen	HetSUS	0
Cass	9	2	Chlorimuron-ethyl + fomesafen	HetSUS	0
Cass	10	2	Chlorimuron-ethyl + fomesafen	RESSUS	0
Cass	1	1	Chlorimuron-ethyl + glyphosate	HetH	100
Cass	2	1	Chlorimuron-ethyl + glyphosate	SUSH	100
Cass	3	1	Chlorimuron-ethyl + glyphosate	SUSH	0
Cass	4	1	Chlorimuron-ethyl + glyphosate	HetH	0
Cass	5	1	Chlorimuron-ethyl + glyphosate	HetH	100
Cass	6	1	Chlorimuron-ethyl + glyphosate	SUSH	100
Cass	7	1	Chlorimuron-ethyl + glyphosate	HetH	0
Cass	8	1	Chlorimuron-ethyl + glyphosate	SUSH	0
Cass	9	1	Chlorimuron-ethyl + glyphosate	SUSH	100

Cass	10	1	Chlorimuron-ethyl + glyphosate	SUSH	100
Cass	1	2	Chlorimuron-ethyl + glyphosate	RESH	100
Cass	2	2	Chlorimuron-ethyl + glyphosate	RESH	100
Cass	3	2	Chlorimuron-ethyl + glyphosate	HetH	100
Cass	4	2	Chlorimuron-ethyl + glyphosate	SUSH	100
Cass	5	2	Chlorimuron-ethyl + glyphosate	SUSH	0
Cass	6	2	Chlorimuron-ethyl + glyphosate	HetH	100
Cass	7	2	Chlorimuron-ethyl + glyphosate	RESH	100
Cass	8	2	Chlorimuron-ethyl + glyphosate	RESH	100
Cass	9	2	Chlorimuron-ethyl + glyphosate	HetH	100
Cass	10	2	Chlorimuron-ethyl + glyphosate	RESH	100
Cass	1	1	Fomesafen + glyphosate	SUSH	0
Cass	2	1	Fomesafen + glyphosate	SUSH	0
Cass	3	1	Fomesafen + glyphosate	SUSH	0
Cass	4	1	Fomesafen + glyphosate	SUSH	0
Cass	5	1	Fomesafen + glyphosate	SUSH	0
Cass	6	1	Fomesafen + glyphosate	SUSH	0
Cass	7	1	Fomesafen + glyphosate	SUSH	0
Cass	8	1	Fomesafen + glyphosate	SUSH	0
Cass	9	1	Fomesafen + glyphosate	SUSH	0
Cass	10	1	Fomesafen + glyphosate	SUSH	0
Cass	1	2	Fomesafen + glyphosate	SUSH	0
Cass	2	2	Fomesafen + glyphosate	SUSH	0
Cass	3	2	Fomesafen + glyphosate	SUSH	0
Cass	4	2	Fomesafen + glyphosate	SUSH	0
Cass	5	2	Fomesafen + glyphosate	SUSH	0
Cass	6	2	Fomesafen + glyphosate	SUSH	0
Cass	7	2	Fomesafen + glyphosate	SUSH	0
Cass	8	2	Fomesafen + glyphosate	SUSH	0
Cass	9	2	Fomesafen + glyphosate	SUSH	0
Cass	10	2	Fomesafen + glyphosate	SUSH	0
Cass	1	1	Chlorimuron-ethyl + fomesafen + glyphosate	HetSUSH	0
Cass	2	1	Chlorimuron-ethyl + fomesafen + glyphosate	SUSSUSH	0
Cass	3	1	Chlorimuron-ethyl + fomesafen + glyphosate	HetSUSH	0
Cass	4	1	Chlorimuron-ethyl + fomesafen + glyphosate	HetSUSH	0
Cass	5	1	Chlorimuron-ethyl + fomesafen + glyphosate	RESSUSH	0
Cass	6	1	Chlorimuron-ethyl + fomesafen + glyphosate	HetSUSH	0
Cass	7	1	Chlorimuron-ethyl + fomesafen + glyphosate	HetSUSH	0
Cass	8	1	Chlorimuron-ethyl + fomesafen + glyphosate	SUSSUSH	0
Cass	9	1	Chlorimuron-ethyl + fomesafen + glyphosate	HetSUSH	0
Cass	10	1	Chlorimuron-ethyl + fomesafen + glyphosate	HetSUSH	0
Cass	1	2	Chlorimuron-ethyl + fomesafen + glyphosate	SUSSUSH	0
Cass	2	2	Chlorimuron-ethyl + fomesafen + glyphosate	HetSUSH	0
Cass	3	2	Chlorimuron-ethyl + fomesafen + glyphosate	HetSUSH	0
Cass	4	2	Chlorimuron-ethyl + fomesafen + glyphosate	HetSUSH	0
Cass	5	2	Chlorimuron-ethyl + fomesafen + glyphosate	HetSUSH	0
Cass	6	2	Chlorimuron-ethyl + fomesafen + glyphosate	SUSSUSH	0

Cass	7	2	Chlorimuron-ethyl + fomesafen + glyphosate	HetSUSH	0
Cass	8	2	Chlorimuron-ethyl + fomesafen + glyphosate	HetSUSH	0
Cass	9	2	Chlorimuron-ethyl + fomesafen + glyphosate	RESSUSH	0
Cass	10	2	Chlorimuron-ethyl + fomesafen + glyphosate	RESSUSH	0
Washington	1	1	Chlorimuron-ethyl	SUS	0
Washington	2	1	Chlorimuron-ethyl	SUS	100
Washington	3	1	Chlorimuron-ethyl	SUS	100
Washington	4	1	Chlorimuron-ethyl	SUS	0
Washington	5	1	Chlorimuron-ethyl	SUS	0
Washington	6	1	Chlorimuron-ethyl	SUS	0
Washington	7	1	Chlorimuron-ethyl	SUS	0
Washington	8	1	Chlorimuron-ethyl	SUS	0
Washington	9	1	Chlorimuron-ethyl	SUS	0
Washington	10	1	Chlorimuron-ethyl	SUS	0
Washington	1	2	Chlorimuron-ethyl	SUS	100
Washington	2	2	Chlorimuron-ethyl	SUS	0
Washington	3	2	Chlorimuron-ethyl	SUS	0
Washington	4	2	Chlorimuron-ethyl	SUS	100
Washington	5	2	Chlorimuron-ethyl	SUS	0
Washington	6	2	Chlorimuron-ethyl	SUS	0
Washington	7	2	Chlorimuron-ethyl	SUS	100
Washington	8	2	Chlorimuron-ethyl	SUS	0
Washington	9	2	Chlorimuron-ethyl	SUS	0
Washington	10	2	Chlorimuron-ethyl	SUS	0
Washington	1	1	Fomesafen	SUS	0
Washington	2	1	Fomesafen	SUS	0
Washington	3	1	Fomesafen	SUS	0
Washington	4	1	Fomesafen	SUS	0
Washington	5	1	Fomesafen	SUS	0
Washington	6	1	Fomesafen	SUS	0
Washington	7	1	Fomesafen	SUS	0
Washington	8	1	Fomesafen	SUS	0
Washington	9	1	Fomesafen	SUS	0
Washington	10	1	Fomesafen	SUS	0
Washington	1	2	Fomesafen	SUS	0
Washington	2	2	Fomesafen	SUS	0
Washington	3	2	Fomesafen	SUS	0
Washington	4	2	Fomesafen	SUS	0
Washington	5	2	Fomesafen	SUS	0
Washington	6	2	Fomesafen	SUS	0
Washington	7	2	Fomesafen	SUS	0
Washington	8	2	Fomesafen	SUS	0
Washington	9	2	Fomesafen	SUS	0
Washington	10	2	Fomesafen	SUS	0
Washington	1	1	Glyphosate	L	0
Washington	2	1	Glyphosate	L	0
Washington	3	1	Glyphosate	L	0

Washington	4	1	Glyphosate	L	0
Washington	5	1	Glyphosate	L	0
Washington	6	1	Glyphosate	L	0
Washington	7	1	Glyphosate	L	0
Washington	8	1	Glyphosate	L	0
Washington	9	1	Glyphosate	L	0
Washington	10	1	Glyphosate	L	0
Washington	1	2	Glyphosate	L	0
Washington	2	2	Glyphosate	L	0
Washington	3	2	Glyphosate	L	0
Washington	4	2	Glyphosate	L	0
Washington	5	2	Glyphosate	L	0
Washington	6	2	Glyphosate	L	0
Washington	7	2	Glyphosate	L	0
Washington	8	2	Glyphosate	L	0
Washington	9	2	Glyphosate	L	0
Washington	10	2	Glyphosate	L	0
Washington	1	1	Chlorimuron-ethyl + fomesafen	SUSSUS	0
Washington	2	1	Chlorimuron-ethyl + fomesafen	SUSSUS	0
Washington	3	1	Chlorimuron-ethyl + fomesafen	SUSSUS	0
Washington	4	1	Chlorimuron-ethyl + fomesafen	SUSSUS	0
Washington	5	1	Chlorimuron-ethyl + fomesafen	SUSSUS	0
Washington	6	1	Chlorimuron-ethyl + fomesafen	SUSSUS	0
Washington	7	1	Chlorimuron-ethyl + fomesafen	SUSSUS	0
Washington	8	1	Chlorimuron-ethyl + fomesafen	SUSSUS	0
Washington	9	1	Chlorimuron-ethyl + fomesafen	SUSSUS	0
Washington	10	1	Chlorimuron-ethyl + fomesafen	SUSSUS	0
Washington	1	2	Chlorimuron-ethyl + fomesafen	SUSSUS	0
Washington	2	2	Chlorimuron-ethyl + fomesafen	SUSSUS	0
Washington	3	2	Chlorimuron-ethyl + fomesafen	SUSSUS	0
Washington	4	2	Chlorimuron-ethyl + fomesafen	SUSSUS	0
Washington	5	2	Chlorimuron-ethyl + fomesafen	SUSSUS	0
Washington	6	2	Chlorimuron-ethyl + fomesafen	SUSSUS	0
Washington	7	2	Chlorimuron-ethyl + fomesafen	SUSSUS	0
Washington	8	2	Chlorimuron-ethyl + fomesafen	SUSSUS	0
Washington	9	2	Chlorimuron-ethyl + fomesafen	SUSSUS	0
Washington	10	2	Chlorimuron-ethyl + fomesafen	SUSSUS	0
Washington	1	1	Chlorimuron-ethyl + glyphosate	SUSL	0
Washington	2	1	Chlorimuron-ethyl + glyphosate	SUSL	0
Washington	3	1	Chlorimuron-ethyl + glyphosate	SUSL	0
Washington	4	1	Chlorimuron-ethyl + glyphosate	SUSL	0
Washington	5	1	Chlorimuron-ethyl + glyphosate	SUSL	0
Washington	6	1	Chlorimuron-ethyl + glyphosate	SUSL	0
Washington	7	1	Chlorimuron-ethyl + glyphosate	SUSL	0
Washington	8	1	Chlorimuron-ethyl + glyphosate	SUSL	0
Washington	9	1	Chlorimuron-ethyl + glyphosate	SUSL	0
Washington	10	1	Chlorimuron-ethyl + glyphosate	SUSL	0

Washington	1	2	Chlorimuron-ethyl + glyphosate	SUSL	0
Washington	2	2	Chlorimuron-ethyl + glyphosate	SUSL	0
Washington	3	2	Chlorimuron-ethyl + glyphosate	SUSL	0
Washington	4	2	Chlorimuron-ethyl + glyphosate	SUSL	0
Washington	5	2	Chlorimuron-ethyl + glyphosate	SUSL	0
Washington	6	2	Chlorimuron-ethyl + glyphosate	SUSL	0
Washington	7	2	Chlorimuron-ethyl + glyphosate	SUSL	0
Washington	8	2	Chlorimuron-ethyl + glyphosate	SUSL	0
Washington	9	2	Chlorimuron-ethyl + glyphosate	SUSL	0
Washington	10	2	Chlorimuron-ethyl + glyphosate	SUSL	0
Washington	1	1	Fomesafen + glyphosate	SUSL	0
Washington	2	1	Fomesafen + glyphosate	SUSL	0
Washington	3	1	Fomesafen + glyphosate	SUSL	0
Washington	4	1	Fomesafen + glyphosate	SUSL	0
Washington	5	1	Fomesafen + glyphosate	SUSL	0
Washington	6	1	Fomesafen + glyphosate	SUSL	0
Washington	7	1	Fomesafen + glyphosate	SUSL	0
Washington	8	1	Fomesafen + glyphosate	SUSL	0
Washington	9	1	Fomesafen + glyphosate	SUSL	0
Washington	10	1	Fomesafen + glyphosate	SUSL	0
Washington	1	2	Fomesafen + glyphosate	SUSL	0
Washington	2	2	Fomesafen + glyphosate	SUSL	0
Washington	3	2	Fomesafen + glyphosate	SUSL	0
Washington	4	2	Fomesafen + glyphosate	SUSL	0
Washington	5	2	Fomesafen + glyphosate	SUSL	0
Washington	6	2	Fomesafen + glyphosate	SUSL	0
Washington	7	2	Fomesafen + glyphosate	SUSL	0
Washington	8	2	Fomesafen + glyphosate	SUSL	0
Washington	9	2	Fomesafen + glyphosate	SUSL	0
Washington	10	2	Fomesafen + glyphosate	SUSL	0
Washington	1	1	Chlorimuron-ethyl + fomesafen + glyphosate	SUSSUSL	0
Washington	2	1	Chlorimuron-ethyl + fomesafen + glyphosate	SUSSUSL	0
Washington	3	1	Chlorimuron-ethyl + fomesafen + glyphosate	SUSSUSL	0
Washington	4	1	Chlorimuron-ethyl + fomesafen + glyphosate	SUSSUSL	0
Washington	5	1	Chlorimuron-ethyl + fomesafen + glyphosate	SUSSUSL	0
Washington	6	1	Chlorimuron-ethyl + fomesafen + glyphosate	SUSSUSL	0
Washington	7	1	Chlorimuron-ethyl + fomesafen + glyphosate	SUSSUSL	0
Washington	8	1	Chlorimuron-ethyl + fomesafen + glyphosate	SUSSUSL	0
Washington	9	1	Chlorimuron-ethyl + fomesafen + glyphosate	SUSSUSL	0
Washington	10	1	Chlorimuron-ethyl + fomesafen + glyphosate	SUSSUSL	0
Washington	1	2	Chlorimuron-ethyl + fomesafen + glyphosate	SUSSUSL	0
Washington	2	2	Chlorimuron-ethyl + fomesafen + glyphosate	SUSSUSL	0
Washington	3	2	Chlorimuron-ethyl + fomesafen + glyphosate	SUSSUSL	0
Washington	4	2	Chlorimuron-ethyl + fomesafen + glyphosate	SUSSUSL	0
Washington	5	2	Chlorimuron-ethyl + fomesafen + glyphosate	SUSSUSL	0
Washington	6	2	Chlorimuron-ethyl + fomesafen + glyphosate	SUSSUSL	0
Washington	7	2	Chlorimuron-ethyl + fomesafen + glyphosate	SUSSUSL	0

Washington	8	2	Chlorimuron-ethyl + fomesafen + glyphosate	SUSSUSL	0
Washington	9	2	Chlorimuron-ethyl + fomesafen + glyphosate	SUSSUSL	0
Washington	10	2	Chlorimuron-ethyl + fomesafen + glyphosate	SUSSUSL	0
Daviess	1	1	Chlorimuron-ethyl	SUS	0
Daviess	2	1	Chlorimuron-ethyl	SUS	0
Daviess	3	1	Chlorimuron-ethyl	SUS	100
Daviess	4	1	Chlorimuron-ethyl	SUS	100
Daviess	5	1	Chlorimuron-ethyl	SUS	100
Daviess	6	1	Chlorimuron-ethyl	SUS	100
Daviess	7	1	Chlorimuron-ethyl	Heterozygote	100
Daviess	8	1	Chlorimuron-ethyl	SUS	0
Daviess	9	1	Chlorimuron-ethyl	Heterozygote	100
Daviess	10	1	Chlorimuron-ethyl	SUS	0
Daviess	11	1	Chlorimuron-ethyl	SUS	100
Daviess	12	1	Chlorimuron-ethyl	SUS	100
Daviess	13	1	Chlorimuron-ethyl	Heterozygote	100
Daviess	14	1	Chlorimuron-ethyl	SUS	0
Daviess	15	1	Chlorimuron-ethyl	SUS	0
Daviess	16	1	Chlorimuron-ethyl	Heterozygote	100
Daviess	17	1	Chlorimuron-ethyl	SUS	100
Daviess	18	1	Chlorimuron-ethyl	SUS	100
Daviess	19	1	Chlorimuron-ethyl	SUS	0
Daviess	20	1	Chlorimuron-ethyl	SUS	100
Daviess	1	2	Chlorimuron-ethyl	SUS	0
Daviess	2	2	Chlorimuron-ethyl	SUS	0
Daviess	3	2	Chlorimuron-ethyl	SUS	100
Daviess	4	2	Chlorimuron-ethyl	Heterozygote	100
Daviess	5	2	Chlorimuron-ethyl	SUS	100
Daviess	6	2	Chlorimuron-ethyl	SUS	100
Daviess	7	2	Chlorimuron-ethyl	RES	100
Daviess	8	2	Chlorimuron-ethyl	SUS	100
Daviess	9	2	Chlorimuron-ethyl	SUS	0
Daviess	10	2	Chlorimuron-ethyl	Heterozygote	100
Daviess	11	2	Chlorimuron-ethyl	SUS	100
Daviess	12	2	Chlorimuron-ethyl	SUS	100
Daviess	13	2	Chlorimuron-ethyl	SUS	100
Daviess	14	2	Chlorimuron-ethyl	SUS	100
Daviess	15	2	Chlorimuron-ethyl	Heterozygote	100
Daviess	16	2	Chlorimuron-ethyl	Heterozygote	100
Daviess	17	2	Chlorimuron-ethyl	Heterozygote	100
Daviess	18	2	Chlorimuron-ethyl	Heterozygote	100
Daviess	19	2	Chlorimuron-ethyl	SUS	0
Daviess	20	2	Chlorimuron-ethyl	SUS	0
Daviess	1	1	Fomesafen	Heterozygote	0
Daviess	2	1	Fomesafen	SUS	0
Daviess	3	1	Fomesafen	SUS	0
Daviess	4	1	Fomesafen	Heterozygote	100

Daviess	5	1	Fomesafen	Heterozygote	0
Daviess	6	1	Fomesafen	SUS	0
Daviess	7	1	Fomesafen	SUS	0
Daviess	8	1	Fomesafen	SUS	0
Daviess	9	1	Fomesafen	SUS	0
Daviess	10	1	Fomesafen	SUS	0
Daviess	11	1	Fomesafen	SUS	0
Daviess	12	1	Fomesafen	SUS	0
Daviess	13	1	Fomesafen	Heterozygote	100
Daviess	14	1	Fomesafen	SUS	0
Daviess	15	1	Fomesafen	RES	100
Daviess	16	1	Fomesafen	Heterozygote	100
Daviess	17	1	Fomesafen	SUS	0
Daviess	18	1	Fomesafen	RES	100
Daviess	19	1	Fomesafen	SUS	0
Daviess	20	1	Fomesafen	SUS	0
Daviess	1	2	Fomesafen	Heterozygote	100
Daviess	2	2	Fomesafen	SUS	0
Daviess	3	2	Fomesafen	SUS	0
Daviess	4	2	Fomesafen	Heterozygote	0
Daviess	5	2	Fomesafen	SUS	0
Daviess	6	2	Fomesafen	Heterozygote	0
Daviess	7	2	Fomesafen	SUS	0
Daviess	8	2	Fomesafen	Heterozygote	0
Daviess	9	2	Fomesafen	Heterozygote	0
Daviess	10	2	Fomesafen	SUS	0
Daviess	11	2	Fomesafen	SUS	0
Daviess	12	2	Fomesafen	SUS	0
Daviess	13	2	Fomesafen	RES	100
Daviess	14	2	Fomesafen	Heterozygote	100
Daviess	15	2	Fomesafen	SUS	0
Daviess	16	2	Fomesafen	SUS	0
Daviess	17	2	Fomesafen	Heterozygote	100
Daviess	18	2	Fomesafen	SUS	0
Daviess	19	2	Fomesafen	SUS	0
Daviess	20	2	Fomesafen	SUS	0
Daviess	1	1	Glyphosate	H	100
Daviess	2	1	Glyphosate	H	100
Daviess	3	1	Glyphosate	H	0
Daviess	4	1	Glyphosate	H	0
Daviess	5	1	Glyphosate	H	100
Daviess	6	1	Glyphosate	H	0
Daviess	7	1	Glyphosate	H	100
Daviess	8	1	Glyphosate	H	0
Daviess	9	1	Glyphosate	H	0
Daviess	10	1	Glyphosate	H	100
Daviess	11	1	Glyphosate	H	0

Daviess	12	1	Glyphosate	H	0
Daviess	13	1	Glyphosate	H	0
Daviess	14	1	Glyphosate	H	0
Daviess	15	1	Glyphosate	H	0
Daviess	16	1	Glyphosate	H	100
Daviess	17	1	Glyphosate	H	100
Daviess	18	1	Glyphosate	H	100
Daviess	19	1	Glyphosate	H	100
Daviess	20	1	Glyphosate	H	100
Daviess	1	2	Glyphosate	H	0
Daviess	2	2	Glyphosate	H	0
Daviess	3	2	Glyphosate	H	0
Daviess	4	2	Glyphosate	H	0
Daviess	5	2	Glyphosate	H	0
Daviess	6	2	Glyphosate	H	0
Daviess	7	2	Glyphosate	H	0
Daviess	8	2	Glyphosate	H	100
Daviess	9	2	Glyphosate	H	100
Daviess	10	2	Glyphosate	H	100
Daviess	11	2	Glyphosate	H	0
Daviess	12	2	Glyphosate	H	0
Daviess	13	2	Glyphosate	H	100
Daviess	14	2	Glyphosate	H	0
Daviess	15	2	Glyphosate	H	100
Daviess	16	2	Glyphosate	H	100
Daviess	17	2	Glyphosate	H	100
Daviess	18	2	Glyphosate	H	0
Daviess	19	2	Glyphosate	H	100
Daviess	20	2	Glyphosate	H	0
Daviess	1	1	Chlorimuron-ethyl + fomesafen	SUSSUS	0
Daviess	2	1	Chlorimuron-ethyl + fomesafen	SUSSUS	0
Daviess	3	1	Chlorimuron-ethyl + fomesafen	SUSHet	0
Daviess	4	1	Chlorimuron-ethyl + fomesafen	SUSSUS	0
Daviess	5	1	Chlorimuron-ethyl + fomesafen	HetHet	100
Daviess	6	1	Chlorimuron-ethyl + fomesafen	HetHet	0
Daviess	7	1	Chlorimuron-ethyl + fomesafen	SUSSUS	0
Daviess	8	1	Chlorimuron-ethyl + fomesafen	SUSSUS	0
Daviess	9	1	Chlorimuron-ethyl + fomesafen	SUSSUS	0
Daviess	10	1	Chlorimuron-ethyl + fomesafen	HetHet	0
Daviess	11	1	Chlorimuron-ethyl + fomesafen	SUSSUS	0
Daviess	12	1	Chlorimuron-ethyl + fomesafen	SUSSUS	0
Daviess	13	1	Chlorimuron-ethyl + fomesafen	SUSSUS	0
Daviess	14	1	Chlorimuron-ethyl + fomesafen	HetHet	0
Daviess	15	1	Chlorimuron-ethyl + fomesafen	SUSSUS	0
Daviess	16	1	Chlorimuron-ethyl + fomesafen	HetRES	0
Daviess	17	1	Chlorimuron-ethyl + fomesafen	SUSSUS	0
Daviess	18	1	Chlorimuron-ethyl + fomesafen	HetHet	100

Daviess	19	1	Chlorimuron-ethyl + fomesafen	SUSSUS	0
Daviess	20	1	Chlorimuron-ethyl + fomesafen	HetHet	100
Daviess	1	2	Chlorimuron-ethyl + fomesafen	HetSUS	0
Daviess	2	2	Chlorimuron-ethyl + fomesafen	SUSHet	100
Daviess	3	2	Chlorimuron-ethyl + fomesafen	SUSSUS	0
Daviess	4	2	Chlorimuron-ethyl + fomesafen	SUSRES	0
Daviess	5	2	Chlorimuron-ethyl + fomesafen	HetHet	100
Daviess	6	2	Chlorimuron-ethyl + fomesafen	RESRES	100
Daviess	7	2	Chlorimuron-ethyl + fomesafen	SUSSUS	0
Daviess	8	2	Chlorimuron-ethyl + fomesafen	HetHet	0
Daviess	9	2	Chlorimuron-ethyl + fomesafen	HetHet	100
Daviess	10	2	Chlorimuron-ethyl + fomesafen	SUSSUS	0
Daviess	11	2	Chlorimuron-ethyl + fomesafen	HetHet	100
Daviess	12	2	Chlorimuron-ethyl + fomesafen	SUSSUS	0
Daviess	13	2	Chlorimuron-ethyl + fomesafen	HetHet	0
Daviess	14	2	Chlorimuron-ethyl + fomesafen	SUSSUS	0
Daviess	15	2	Chlorimuron-ethyl + fomesafen	SUSHet	0
Daviess	16	2	Chlorimuron-ethyl + fomesafen	HetSUS	0
Daviess	17	2	Chlorimuron-ethyl + fomesafen	RESHet	0
Daviess	18	2	Chlorimuron-ethyl + fomesafen	HetHet	100
Daviess	19	2	Chlorimuron-ethyl + fomesafen	SUSHet	0
Daviess	20	2	Chlorimuron-ethyl + fomesafen	RESSUS	0
Daviess	1	1	Chlorimuron-ethyl + glyphosate	SUSH	0
Daviess	2	1	Chlorimuron-ethyl + glyphosate	SUSH	100
Daviess	3	1	Chlorimuron-ethyl + glyphosate	SUSH	100
Daviess	4	1	Chlorimuron-ethyl + glyphosate	SUSH	0
Daviess	5	1	Chlorimuron-ethyl + glyphosate	SUSH	100
Daviess	6	1	Chlorimuron-ethyl + glyphosate	HetH	0
Daviess	7	1	Chlorimuron-ethyl + glyphosate	HetH	100
Daviess	8	1	Chlorimuron-ethyl + glyphosate	SUSH	0
Daviess	9	1	Chlorimuron-ethyl + glyphosate	SUSH	0
Daviess	10	1	Chlorimuron-ethyl + glyphosate	RESH	100
Daviess	11	1	Chlorimuron-ethyl + glyphosate	SUSH	0
Daviess	12	1	Chlorimuron-ethyl + glyphosate	SUSH	0
Daviess	13	1	Chlorimuron-ethyl + glyphosate	SUSH	0
Daviess	14	1	Chlorimuron-ethyl + glyphosate	HetH	0
Daviess	15	1	Chlorimuron-ethyl + glyphosate	SUSH	100
Daviess	16	1	Chlorimuron-ethyl + glyphosate	RESH	0
Daviess	17	1	Chlorimuron-ethyl + glyphosate	HetH	0
Daviess	18	1	Chlorimuron-ethyl + glyphosate	SUSH	0
Daviess	19	1	Chlorimuron-ethyl + glyphosate	HetH	0
Daviess	20	1	Chlorimuron-ethyl + glyphosate	RESH	100
Daviess	1	2	Chlorimuron-ethyl + glyphosate	SUSH	0
Daviess	2	2	Chlorimuron-ethyl + glyphosate	SUSH	0
Daviess	3	2	Chlorimuron-ethyl + glyphosate	SUSH	0
Daviess	4	2	Chlorimuron-ethyl + glyphosate	SUSH	100
Daviess	5	2	Chlorimuron-ethyl + glyphosate	SUSH	0

Daviess	6	2	Chlorimuron-ethyl + glyphosate	RESH	0
Daviess	7	2	Chlorimuron-ethyl + glyphosate	HetH	0
Daviess	8	2	Chlorimuron-ethyl + glyphosate	SUSH	0
Daviess	9	2	Chlorimuron-ethyl + glyphosate	SUSH	100
Daviess	10	2	Chlorimuron-ethyl + glyphosate	HetH	0
Daviess	11	2	Chlorimuron-ethyl + glyphosate	SUSH	0
Daviess	12	2	Chlorimuron-ethyl + glyphosate	SUSH	100
Daviess	13	2	Chlorimuron-ethyl + glyphosate	SUSH	0
Daviess	14	2	Chlorimuron-ethyl + glyphosate	SUSH	100
Daviess	15	2	Chlorimuron-ethyl + glyphosate	SUSH	0
Daviess	16	2	Chlorimuron-ethyl + glyphosate	HetH	0
Daviess	17	2	Chlorimuron-ethyl + glyphosate	HetH	100
Daviess	18	2	Chlorimuron-ethyl + glyphosate	SUSH	100
Daviess	19	2	Chlorimuron-ethyl + glyphosate	HetH	0
Daviess	20	2	Chlorimuron-ethyl + glyphosate	HetH	0
Daviess	1	1	Fomesafen + glyphosate	SUSH	0
Daviess	2	1	Fomesafen + glyphosate	SUSH	0
Daviess	3	1	Fomesafen + glyphosate	RESH	100
Daviess	4	1	Fomesafen + glyphosate	RESH	100
Daviess	5	1	Fomesafen + glyphosate	SUSH	0
Daviess	6	1	Fomesafen + glyphosate	SUSH	0
Daviess	7	1	Fomesafen + glyphosate	SUSH	0
Daviess	8	1	Fomesafen + glyphosate	HetH	0
Daviess	9	1	Fomesafen + glyphosate	HetH	100
Daviess	10	1	Fomesafen + glyphosate	HetH	0
Daviess	11	1	Fomesafen + glyphosate	HetH	100
Daviess	12	1	Fomesafen + glyphosate	SUSH	0
Daviess	13	1	Fomesafen + glyphosate	HetH	100
Daviess	14	1	Fomesafen + glyphosate	HetH	100
Daviess	15	1	Fomesafen + glyphosate	SUSH	0
Daviess	16	1	Fomesafen + glyphosate	HetH	0
Daviess	17	1	Fomesafen + glyphosate	HetH	0
Daviess	18	1	Fomesafen + glyphosate	SUSH	0
Daviess	19	1	Fomesafen + glyphosate	SUSH	0
Daviess	20	1	Fomesafen + glyphosate	HetH	100
Daviess	1	2	Fomesafen + glyphosate	HetH	0
Daviess	2	2	Fomesafen + glyphosate	SUSH	0
Daviess	3	2	Fomesafen + glyphosate	HetH	0
Daviess	4	2	Fomesafen + glyphosate	HetH	0
Daviess	5	2	Fomesafen + glyphosate	SUSH	0
Daviess	6	2	Fomesafen + glyphosate	RESH	100
Daviess	7	2	Fomesafen + glyphosate	HetH	100
Daviess	8	2	Fomesafen + glyphosate	SUSH	0
Daviess	9	2	Fomesafen + glyphosate	SUSH	0
Daviess	10	2	Fomesafen + glyphosate	SUSH	0
Daviess	11	2	Fomesafen + glyphosate	SUSH	0
Daviess	12	2	Fomesafen + glyphosate	SUSH	0

Daviess	13	2	Fomesafen + glyphosate	SUSH	0
Daviess	14	2	Fomesafen + glyphosate	HetH	100
Daviess	15	2	Fomesafen + glyphosate	SUSH	0
Daviess	16	2	Fomesafen + glyphosate	SUSH	0
Daviess	17	2	Fomesafen + glyphosate	SUSH	0
Daviess	18	2	Fomesafen + glyphosate	SUSH	0
Daviess	19	2	Fomesafen + glyphosate	RESH	100
Daviess	20	2	Fomesafen + glyphosate	HetH	0
Daviess	1	1	Chlorimuron-ethyl + fomesafen + glyphosate	HetHetH	0
Daviess	2	1	Chlorimuron-ethyl + fomesafen + glyphosate	SUSSUSH	0
Daviess	3	1	Chlorimuron-ethyl + fomesafen + glyphosate	SUSSUSH	0
Daviess	4	1	Chlorimuron-ethyl + fomesafen + glyphosate	SUSHetL	0
Daviess	5	1	Chlorimuron-ethyl + fomesafen + glyphosate	HetRESH	100
Daviess	6	1	Chlorimuron-ethyl + fomesafen + glyphosate	SUSSUSH	100
Daviess	7	1	Chlorimuron-ethyl + fomesafen + glyphosate	SUSSUSH	0
Daviess	8	1	Chlorimuron-ethyl + fomesafen + glyphosate	SUSSUSH	0
Daviess	9	1	Chlorimuron-ethyl + fomesafen + glyphosate	RESRESH	100
Daviess	10	1	Chlorimuron-ethyl + fomesafen + glyphosate	SUSHetH	0
Daviess	11	1	Chlorimuron-ethyl + fomesafen + glyphosate	SUSHetH	0
Daviess	12	1	Chlorimuron-ethyl + fomesafen + glyphosate	HetRESH	100
Daviess	13	1	Chlorimuron-ethyl + fomesafen + glyphosate	HetSUSH	0
Daviess	14	1	Chlorimuron-ethyl + fomesafen + glyphosate	SUSSUSH	0
Daviess	15	1	Chlorimuron-ethyl + fomesafen + glyphosate	SUSSUSH	0
Daviess	16	1	Chlorimuron-ethyl + fomesafen + glyphosate	SUSSUSH	0
Daviess	17	1	Chlorimuron-ethyl + fomesafen + glyphosate	HetSUSH	0
Daviess	18	1	Chlorimuron-ethyl + fomesafen + glyphosate	HetHetH	100
Daviess	19	1	Chlorimuron-ethyl + fomesafen + glyphosate	HetRESH	100
Daviess	20	1	Chlorimuron-ethyl + fomesafen + glyphosate	SUSSUSH	0
Daviess	1	2	Chlorimuron-ethyl + fomesafen + glyphosate	SUSSUSH	0
Daviess	2	2	Chlorimuron-ethyl + fomesafen + glyphosate	HetSUSH	100
Daviess	3	2	Chlorimuron-ethyl + fomesafen + glyphosate	SUSHetH	100
Daviess	4	2	Chlorimuron-ethyl + fomesafen + glyphosate	HetHetH	0
Daviess	5	2	Chlorimuron-ethyl + fomesafen + glyphosate	HetRESH	100
Daviess	6	2	Chlorimuron-ethyl + fomesafen + glyphosate	SUSSUSH	0
Daviess	7	2	Chlorimuron-ethyl + fomesafen + glyphosate	SUSSUSH	0
Daviess	8	2	Chlorimuron-ethyl + fomesafen + glyphosate	HetHetH	0
Daviess	9	2	Chlorimuron-ethyl + fomesafen + glyphosate	HetSUSH	0
Daviess	10	2	Chlorimuron-ethyl + fomesafen + glyphosate	SUSHetH	0
Daviess	11	2	Chlorimuron-ethyl + fomesafen + glyphosate	SUSHetH	100
Daviess	12	2	Chlorimuron-ethyl + fomesafen + glyphosate	HetHetH	0
Daviess	13	2	Chlorimuron-ethyl + fomesafen + glyphosate	HetSUSH	0
Daviess	14	2	Chlorimuron-ethyl + fomesafen + glyphosate	HetHetH	100
Daviess	15	2	Chlorimuron-ethyl + fomesafen + glyphosate	SUSSUSH	0
Daviess	16	2	Chlorimuron-ethyl + fomesafen + glyphosate	SUSHetH	0
Daviess	17	2	Chlorimuron-ethyl + fomesafen + glyphosate	SUSSUSH	0
Daviess	18	2	Chlorimuron-ethyl + fomesafen + glyphosate	SUSHetH	100
Daviess	19	2	Chlorimuron-ethyl + fomesafen + glyphosate	HetHetH	100

Daviess	20	2	Chlorimuron-ethyl + fomesafen + glyphosate	SUSHetH	100
Unknown	1	1	Chlorimuron-ethyl	Heterozygote	100
Unknown	2	1	Chlorimuron-ethyl	SUS	100
Unknown	3	1	Chlorimuron-ethyl	Heterozygote	100
Unknown	4	1	Chlorimuron-ethyl	SUS	0
Unknown	5	1	Chlorimuron-ethyl	Heterozygote	100
Unknown	6	1	Chlorimuron-ethyl	Heterozygote	100
Unknown	7	1	Chlorimuron-ethyl	Heterozygote	100
Unknown	8	1	Chlorimuron-ethyl	SUS	100
Unknown	9	1	Chlorimuron-ethyl	Heterozygote	100
Unknown	10	1	Chlorimuron-ethyl	SUS	100
Unknown	1	2	Chlorimuron-ethyl	SUS	100
Unknown	2	2	Chlorimuron-ethyl	Heterozygote	100
Unknown	3	2	Chlorimuron-ethyl	SUS	100
Unknown	4	2	Chlorimuron-ethyl	Heterozygote	100
Unknown	5	2	Chlorimuron-ethyl	SUS	100
Unknown	6	2	Chlorimuron-ethyl	SUS	100
Unknown	7	2	Chlorimuron-ethyl	Heterozygote	100
Unknown	8	2	Chlorimuron-ethyl	Heterozygote	100
Unknown	9	2	Chlorimuron-ethyl	Heterozygote	100
Unknown	10	2	Chlorimuron-ethyl	SUS	100
Unknown	1	1	Fomesafen	SUS	0
Unknown	2	1	Fomesafen	SUS	0
Unknown	3	1	Fomesafen	SUS	0
Unknown	4	1	Fomesafen	SUS	0
Unknown	5	1	Fomesafen	SUS	0
Unknown	6	1	Fomesafen	SUS	0
Unknown	7	1	Fomesafen	SUS	0
Unknown	8	1	Fomesafen	SUS	0
Unknown	9	1	Fomesafen	SUS	0
Unknown	10	1	Fomesafen	SUS	0
Unknown	1	2	Fomesafen	SUS	0
Unknown	2	2	Fomesafen	SUS	0
Unknown	3	2	Fomesafen	SUS	0
Unknown	4	2	Fomesafen	SUS	0
Unknown	5	2	Fomesafen	SUS	0
Unknown	6	2	Fomesafen	SUS	0
Unknown	7	2	Fomesafen	SUS	0
Unknown	8	2	Fomesafen	SUS	0
Unknown	9	2	Fomesafen	SUS	0
Unknown	10	2	Fomesafen	SUS	0
Unknown	1	1	Glyphosate	L	0
Unknown	2	1	Glyphosate	L	0
Unknown	3	1	Glyphosate	L	0
Unknown	4	1	Glyphosate	L	0
Unknown	5	1	Glyphosate	L	0
Unknown	6	1	Glyphosate	L	0

Unknown	7	1	Glyphosate	L	0
Unknown	8	1	Glyphosate	L	0
Unknown	9	1	Glyphosate	L	0
Unknown	10	1	Glyphosate	L	0
Unknown	1	2	Glyphosate	L	0
Unknown	2	2	Glyphosate	L	0
Unknown	3	2	Glyphosate	L	0
Unknown	4	2	Glyphosate	L	0
Unknown	5	2	Glyphosate	L	0
Unknown	6	2	Glyphosate	L	0
Unknown	7	2	Glyphosate	L	0
Unknown	8	2	Glyphosate	L	0
Unknown	9	2	Glyphosate	L	0
Unknown	10	2	Glyphosate	L	0
Unknown	1	1	Chlorimuron-ethyl + fomesafen	SUSSUS	0
Unknown	2	1	Chlorimuron-ethyl + fomesafen	HetSUS	0
Unknown	3	1	Chlorimuron-ethyl + fomesafen	HetSUS	0
Unknown	4	1	Chlorimuron-ethyl + fomesafen	HetSUS	0
Unknown	5	1	Chlorimuron-ethyl + fomesafen	RESSUS	0
Unknown	6	1	Chlorimuron-ethyl + fomesafen	RESSUS	0
Unknown	7	1	Chlorimuron-ethyl + fomesafen	RESSUS	0
Unknown	8	1	Chlorimuron-ethyl + fomesafen	SUSSUS	0
Unknown	9	1	Chlorimuron-ethyl + fomesafen	HetSUS	0
Unknown	10	1	Chlorimuron-ethyl + fomesafen	HetSUS	0
Unknown	1	2	Chlorimuron-ethyl + fomesafen	SUSSUS	0
Unknown	2	2	Chlorimuron-ethyl + fomesafen	SUSSUS	0
Unknown	3	2	Chlorimuron-ethyl + fomesafen	HetSUS	0
Unknown	4	2	Chlorimuron-ethyl + fomesafen	HetSUS	0
Unknown	5	2	Chlorimuron-ethyl + fomesafen	SUSSUS	0
Unknown	6	2	Chlorimuron-ethyl + fomesafen	HetSUS	0
Unknown	7	2	Chlorimuron-ethyl + fomesafen	SUSSUS	0
Unknown	8	2	Chlorimuron-ethyl + fomesafen	HetSUS	0
Unknown	9	2	Chlorimuron-ethyl + fomesafen	RESSUS	0
Unknown	10	2	Chlorimuron-ethyl + fomesafen	HetSUS	0
Unknown	1	1	Chlorimuron-ethyl + glyphosate	SUSL	0
Unknown	2	1	Chlorimuron-ethyl + glyphosate	HetL	0
Unknown	3	1	Chlorimuron-ethyl + glyphosate	SUSL	0
Unknown	4	1	Chlorimuron-ethyl + glyphosate	HetL	0
Unknown	5	1	Chlorimuron-ethyl + glyphosate	SUSL	0
Unknown	6	1	Chlorimuron-ethyl + glyphosate	SUSL	0
Unknown	7	1	Chlorimuron-ethyl + glyphosate	RESL	0
Unknown	8	1	Chlorimuron-ethyl + glyphosate	HetL	0
Unknown	9	1	Chlorimuron-ethyl + glyphosate	SUSL	0
Unknown	10	1	Chlorimuron-ethyl + glyphosate	HetL	0
Unknown	1	2	Chlorimuron-ethyl + glyphosate	SUSL	0
Unknown	2	2	Chlorimuron-ethyl + glyphosate	SUSL	0
Unknown	3	2	Chlorimuron-ethyl + glyphosate	SUSL	0

Unknown	4	2	Chlorimuron-ethyl + glyphosate	HetL	0
Unknown	5	2	Chlorimuron-ethyl + glyphosate	SUSL	0
Unknown	6	2	Chlorimuron-ethyl + glyphosate	RESL	0
Unknown	7	2	Chlorimuron-ethyl + glyphosate	HetL	0
Unknown	8	2	Chlorimuron-ethyl + glyphosate	HetL	0
Unknown	9	2	Chlorimuron-ethyl + glyphosate	HetL	0
Unknown	10	2	Chlorimuron-ethyl + glyphosate	SUSL	0
Unknown	1	1	Fomesafen + glyphosate	SUSL	0
Unknown	2	1	Fomesafen + glyphosate	SUSL	0
Unknown	3	1	Fomesafen + glyphosate	SUSL	0
Unknown	4	1	Fomesafen + glyphosate	SUSL	0
Unknown	5	1	Fomesafen + glyphosate	SUSL	0
Unknown	6	1	Fomesafen + glyphosate	SUSL	0
Unknown	7	1	Fomesafen + glyphosate	SUSL	0
Unknown	8	1	Fomesafen + glyphosate	SUSL	0
Unknown	9	1	Fomesafen + glyphosate	SUSL	0
Unknown	10	1	Fomesafen + glyphosate	SUSL	0
Unknown	1	2	Fomesafen + glyphosate	SUSL	0
Unknown	2	2	Fomesafen + glyphosate	SUSL	0
Unknown	3	2	Fomesafen + glyphosate	SUSL	0
Unknown	4	2	Fomesafen + glyphosate	SUSL	0
Unknown	5	2	Fomesafen + glyphosate	SUSL	0
Unknown	6	2	Fomesafen + glyphosate	SUSL	0
Unknown	7	2	Fomesafen + glyphosate	SUSL	0
Unknown	8	2	Fomesafen + glyphosate	SUSL	0
Unknown	9	2	Fomesafen + glyphosate	SUSL	0
Unknown	10	2	Fomesafen + glyphosate	SUSL	0
Unknown	1	1	Chlorimuron-ethyl + fomesafen + glyphosate	HetSUSL	0
Unknown	2	1	Chlorimuron-ethyl + fomesafen + glyphosate	HetSUSL	0
Unknown	3	1	Chlorimuron-ethyl + fomesafen + glyphosate	HetSUSL	100
Unknown	4	1	Chlorimuron-ethyl + fomesafen + glyphosate	HetSUSL	0
Unknown	5	1	Chlorimuron-ethyl + fomesafen + glyphosate	SUSSUSL	0
Unknown	6	1	Chlorimuron-ethyl + fomesafen + glyphosate	HetSUSL	0
Unknown	7	1	Chlorimuron-ethyl + fomesafen + glyphosate	HetSUSL	0
Unknown	8	1	Chlorimuron-ethyl + fomesafen + glyphosate	SUSSUSL	0
Unknown	9	1	Chlorimuron-ethyl + fomesafen + glyphosate	SUSSUSL	0
Unknown	10	1	Chlorimuron-ethyl + fomesafen + glyphosate	SUSSUSL	0
Unknown	1	2	Chlorimuron-ethyl + fomesafen + glyphosate	SUSSUSL	0
Unknown	2	2	Chlorimuron-ethyl + fomesafen + glyphosate	SUSSUSL	0
Unknown	3	2	Chlorimuron-ethyl + fomesafen + glyphosate	SUSSUSL	0
Unknown	4	2	Chlorimuron-ethyl + fomesafen + glyphosate	HetSUSL	0
Unknown	5	2	Chlorimuron-ethyl + fomesafen + glyphosate	HetSUSL	0
Unknown	6	2	Chlorimuron-ethyl + fomesafen + glyphosate	HetSUSL	0
Unknown	7	2	Chlorimuron-ethyl + fomesafen + glyphosate	SUSSUSL	0
Unknown	8	2	Chlorimuron-ethyl + fomesafen + glyphosate	SUSSUSL	0
Unknown	9	2	Chlorimuron-ethyl + fomesafen + glyphosate	HetSUSL	0
Unknown	10	2	Chlorimuron-ethyl + fomesafen + glyphosate	HetSUSL	0
